# Disparities in integrating non-invasive prenatal testing into antenatal healthcare in Australia: a survey of healthcare professionals

**DOI:** 10.1186/s12884-024-06565-1

**Published:** 2024-05-14

**Authors:** Molly Johnston, Lisa Hui, Hilary Bowman-Smart, Michelle Taylor-Sands, Mark D. Pertile, Catherine Mills

**Affiliations:** 1 Monash Bioethics Centre, Monash University, Wellington Rd, Clayton, 3800 Australia; 2 Department of Obstetrics, Gynaecology and Newborn Health, University of Melbourne, Parkville, 3010 Australia; 3 Reproductive Epidemiology Group, Murdoch Children’s Research Institute, Parkville, 3052 Australia; 4 Mercy Hospital for Women, Heidelberg, 3084 Australia; 5 Northern Health, Epping, 3076 Australia; 6 Australian Centre for Precision Health, University of South Australia, Adelaide, 5061 Australia; 7 Biomedical Ethics Research Group, Murdoch Children’s Research Institute, Parkville, 3052 Australia; 8 Melbourne Law School, University of Melbourne, Carlton, 3053 Australia; 9 Victorian Clinical Genetics Services, Murdoch Children’s Research Institute, Melbourne, VIC 3052 Australia

**Keywords:** Non-invasive prenatal testing, Prenatal screening, Provider experience, Clinical implementation, Equity of access

## Abstract

**Background:**

Non-invasive prenatal testing (NIPT) has been clinically available in Australia on a user-pays basis since 2012. There are numerous providers, with available tests ranging from targeted NIPT (only trisomies 21, 18, and 13 +/- sex chromosome aneuploidy) to genome-wide NIPT. While NIPT is being implemented in the public health care systems of other countries, in Australia, the implementation of NIPT has proceeded without public funding. The aim of this study was to investigate how NIPT has been integrated into antenatal care across Australia and reveal the successes and challenges in its implementation in this context.

**Methods:**

An anonymous online survey was conducted from September to October 2022. Invitations to participate were sent to healthcare professionals (HCPs) involved in the provision of NIPT in Australia through professional society mailing lists and networks. Participants were asked questions on their knowledge of NIPT, delivery of NIPT, and post-test management of results.

**Results:**

A total of 475 HCPs responded, comprising 232 (48.8%) obstetricians, 167 (35.2%) general practitioners, 32 (6.7%) midwives, and 44 (9.3%) genetic specialists. NIPT was most commonly offered as a first-tier test, with most HCPs (*n* = 279; 60.3%) offering it to patients as a choice between NIPT and combined first-trimester screening. Fifty-three percent (*n* = 245) of respondents always offered patients a choice between NIPT for the common autosomal trisomies and expanded (including genome-wide) NIPT. This choice was understood as supporting patient autonomy and informed consent. Cost was seen as a major barrier to access to NIPT, for both targeted and expanded tests. Equitable access, increasing time demands on HCPs, and staying up to date with advances were frequently reported as major challenges in delivering NIPT.

**Conclusions:**

Our findings demonstrate substantial variation in the clinical implementation of NIPT in Australia, including in the offers of expanded screening options. After a decade of clinical use, Australian clinicians still report ongoing challenges in the clinical and equitable provision of NIPT.

**Supplementary Information:**

The online version contains supplementary material available at 10.1186/s12884-024-06565-1.

## Introduction

Since NIPT became available in Australia in 2012, uptake has increased substantially [1]. While other nations such as the Netherlands and Belgium have integrated NIPT into publicly-funded coordinated screening programmes, the delivery of NIPT in Australia has been predominantly provided by commercial laboratories, under the banner of consumer choice [2]. In Australia, antenatal care is accessed through both the public and private sectors, depending on patient preference and financial resources. Some (but not all) medical interventions, tests, and treatments are subsidised through Medicare, Australia’s public healthcare system. Similarly, some antenatal services are reimbursed through private health insurers. Currently, NIPT does not attract a subsidy, with patients required to pay AUD ~ $400-$500 out of pocket to access the test.

Since the first year of NIPT availability, clinicians have been concerned with the financial barriers to access, as NIPT is typically the most expensive prenatal screening test and the only one that does not attract a Medicare subsidy [3]. There are concerns that variation in public funding for prenatal screening in Australia has led to inequities in access to NIPT, particularly for low-income earners and those in remote or rural communities [4, 5]. This conflicts with the stated ethical principles of prenatal screening, including equity of access [6–9].

There are also many clinical controversies in the integration of NIPT into clinical care. The Royal Australian and New Zealand College of Obstetricians and Gynaecologists (RANZCOG) makes a non-prescriptive recommendation that NIPT can be used as a first *or* second-tier screening test depending on “local resources, patient demographics, and individual patient characteristics” [7]. RANZCOG does not endorse routine population-based screening for sex chromosome conditions, genome-wide chromosome abnormalities or microdeletion syndromes, but stops short of proscribing this practice. RANZCOG also recommends the routine use of the 11–13 week nuchal translucency ultrasound alongside NIPT to enable the early detection of fetal structural anomalies. However, it is uncertain how, if at all, clinicians have responded to these recommendations about NIPT. There are also concerns about the increased workload requirements of pretest counselling and the impact on already constrained clinical consultations. How these issues are navigated in clinical practice has implications for what prenatal care is provided to patients and how it is experienced.

A comprehensive view of the contemporary provision of NIPT in Australia is required to assess these concerns. However, there is a lack of clarity about many facets of NIPT delivery in Australia, including who it is offered to, how it is offered, and the challenges to effective provision. While there is some empirical research in the Australian context, these studies were constrained by sample limitations or are no longer relevant to current practice [5, 10–14].

This study aims to investigate how NIPT has been integrated into antenatal care in Australia by examining current practices and views of healthcare professionals (HCPs) involved in NIPT provision. This study sought to identify variabilities in the delivery of care, including the barriers or challenges to the consistent and comprehensive provision of NIPT. An understanding of disparities in care is crucial to improving the delivery of prenatal screening services in Australia.

## Methods

An online survey was conducted in Australia between September and October 2022, using the Qualtrics survey platform. Participants self-selected and were recruited through advertisements on professional society email lists (Royal Australian and New Zealand College of Obstetricians and Gynaecologists; Human Genetics Society of Australasia; Rural Doctors Association of Australia), social media, and snowballing. HCPs who have worked in Australia and been involved in NIPT provision between 2019–2022 were eligible to participate. Eligibility was ascertained through a series of fixed-choice questions at the beginning of the survey. Those deemed ineligible were directed out of the survey.

The survey covered topics such as knowledge of NIPT, test delivery, pre- and post-test counseling, future applications of NIPT, and test data management. The knowledge questions were developed in reference to Lewis [15], extended by input from experts in the investigator team. The survey was developed in consultation with professionals with expertise in obstetrics, clinical genetics, reproductive law, and bioethics. It was piloted on 11 HCPs and questions were refined where necessary. This article reports a subset of the survey data, focusing on HCP knowledge, test uptake and delivery of NIPT, and post-test management of results.

The survey generated both quantitative and qualitative data. Quantitative data were collected to assess and identify trends in attitudes and the practices of the sample. Participants were asked to respond via fixed-choice, Likert scale, and true/false responses. Qualitative data allowed participants to elaborate on their attitudes and experiences, providing richer insights. Categorical data were summarized using frequencies and percentages. Associations between categorical variables were assessed via Chi Square and Fisher’s Exact tests. *P* < 0.05 was considered significant. Analyses were performed using Stata Statistical Software version 17 (StataCorp. 2021. College Station, TX: StataCorp LL).

For comparative analysis, HCPs were allocated to four broad categories:

1. Obstetricians: general obstetrics, fellows, and subspecialties (e.g., ultrasound or maternal fetal medicine specialists);
2. General practitioners (GPs): rural GPs, fellows, trainees, and GPs with further training in office-based or hospital-based obstetrics and gynaecology;
3. Genetic specialists: genetic counselors and clinical geneticists;
4. Midwives.

Qualitative data were analyzed using inductive content analysis [16]. This involves several iterations of coding (categorising segments of data according to their meaning), followed by comparing, grouping, and sub-dividing codes into content categories and subcategories [16]. MJ performed the analysis, and each question was coded by a research assistant, KV (see Acknowledgments), to confirm the coding schema.

## Results

A total of 540 HCPs responded to the survey. Responses with less than 25% completion (*n* = 65) were excluded, resulting in *n* = 475 eligible responses. Participant demographics are reported in Table 1.

**Table 1:** Demographics of the study sample

**Descriptions**	**Total = 475**
	**n (%)**
**Profession**	
Obstetricians	232 (48.8)
General Practitioners	167 (35.2)
Genetic Specialists	44 (9.3)
Midwives	32 (6.7)
**Number of years working in profession**^**a**^	
≤ 5	54 (12.3)
6–15	176 (40.1)
16–25	93 (21.2)
26–35	82 (18.7)
≥ 36	34 (7.7)
**Sector**^**a**^	
Public	148 (33.8)
Private	193 (44.1)
Both public and private	97 (22.1)
**Type of patients predominantly seen**^**a**^	
Public	134 (30.5)
Private	153 (34.9)
Both public and private	152 (34.6)
**Number of patients/year with whom NIPT is discussed**^**a**^	
≤ 5	16 (3.7)
6–20	94 (21.5)
21–50	124 (28.4)
51–100	80 (18.3)
100+	123 (28.1)
**Type of practice/s where they provide NIPT services**^**b**^	
Public hospital or clinic	233 (49.1)
Solo private practice	96 (20.2)
Group private practice	197 (41.5)
Private hospital	15 (3.2)
Other	26 (5.5)
**State/Territory where they provide NIPT services**^**c**^	
Australian Capital Territory	11 (2.9)
New South Wales	91 (23.9)
Northern Territory	4 (1.1)
Queensland	60 (15.8)
South Australia	35 (9.2)
Tasmania	8 (2.1)
Victoria	142 (37.4)
Western Australia	35 (9.2)
**Location of where they provide NIPT services**^**d**^	
Metropolitan	225 (62.8)
Regional	46 (12.8)
Rural	104 (29.1)
**Type of NIPT involvement**	
*Provides information on the use of NIPT*	464 (97.7)
*Provides pre-test counseling and collects consent*	439 (92.4)
*Conveys results following NIPT*	456 (96.0)
*Provides genetic counseling/discusses options following NIPT*	423 (89.1)

^a^Missing data from 36–38 respondents

^b^Missing data from 83 respondents

^c^Missing data from 95 respondents

^d^Rurality was created using postcodes. The reported postcodes were matched to a file [17] which contained a matching rurality for each postcode [column Electorate Rating & Provincial = Regional]. Missing data from 117 respondents

### Offering NIPT and patient choice

Table 2 summarizes how NIPT is being used in antenatal care. The majority of participants provide NIPT as a first-tier test. 60.3% give patients a choice between NIPT and combined first-trimester screening (CFTS), whereas 19% recommend NIPT over CFTS for all patients. Most (86.8%) offer NIPT from 10 weeks gestation.

**Table 2:** The provision of non-invasive prenatal testing (NIPT) in antenatal care, *n* = 475

	**n (%)**
**How NIPT is most commonly offered**	
Offer a choice between NIPT and combined first trimester screening (CFTS) for patients in the first trimester	279 (60.3)
First-tier screening test for all patients	88 (19.0)
First-tier screening test only for patients with higher chance for aneuploidy	12 (2.6)
Second-tier screening test after combined first-trimester screening (CFTS)	49 (10.6)
Other	35 (7.6)
**Gestational age where patients are recommended to undergo NIPT**	
From 9 weeks on	20 (4.3)
From 10 weeks on	400 (86.8)
From 12 weeks on	19 (4.1)
Other	22 (4.8)
**First-trimester ultrasound commonly offered to patients having NIPT as a 1st tier screening test (multiple responses allowed)**	
6–8 weeks (dating)	257 (54.1)
10 weeks (pre-NIPT)	137 (28.8)
11–13 weeks (early fetal structural survey or concurrently with NIPT)	409 (86.1)
Other	24 (5.1)
**Do you offer patients a choice of what to screen for with NIPT**	
Yes	245 (53.0)
No	94 (20.3)
Sometimes	79 (17.1)
N/A to my role	44 (9.5)
**What screening options do you offer patients in addition to trisomies 21, 13, and 18 (multiple responses allowed)**^a^	
Sex chromosome aneuploidies	278 (85.8)
Genome-wide NIPT	93 (28.7)
Microdeletions	102 (31.5)
Single gene disorders	51 (15.7)
Other	18 (5.6)
**Do you refer your patients to one particular brand of NIPT**	
Yes	281 (60.7)
No	125 (27.0)
Sometimes	35 (7.6)
N/A to my role	22 (4.8)
Most common ***reasons for brand choice***^b^ (multiple responses allowed)	
Test performance	137 (28.8)
Convenience of blood collection	128 (26.9)
It is the brand of NIPT with the fastest results	39 (8.2)
It is the brand of NIPT that screens for the most things	36 (7.6)
It is the cheapest	19 (4.0)
It is the easiest to organize	68 (14.3)
It is the brand I am most familiar with	117 (24.6)
It is the preferred brand of the clinic I work at	87 (18.3)
It is the brand recommended by colleagues	49 (10.3)
Clinical support offered by the provider/laboratory	141 (29.7)
It is offered by a not-for-profit organization	49 (10.3)
Education support provided by the provider/laboratory	88 (18.5)
Pre-existing relationship with brand/laboratory	79 (16.6)
Other	47 (9.9)
**Major barriers to access to NIPT for patients** (multiple responses allowed)	
Cost to the patient	447 (94.1)
Access to blood drawing services	29 (6.1)
Patients knowing about the option of the test	150 (31.6)
Healthcare professionals offering the test	127 (26.7)
Informed consent process	107 (22.5)
The time needed to explain the test	101 (21.3)
Other	8 (1.7)

^a^Only asked among those who answered Yes/Sometimes to the question “Do you offer patients a choice of conditions to be screened with NIPT?”; *n* = 324

^b^Only asked among those who answered Yes/Sometimes to the question “Do you usually refer your patients to one particular brand of NIPT?”; *n* = 316

HCPs who worked solely in the public sector were less likely to offer NIPT as a first-tier test for all pregnant patients compared with HCPs who worked solely or partly in private practice. (10.7% vs. 22.9%, respectively; *p* = 0.002). HCPs in the public sector more commonly offer NIPT as a second tier test (21.4% public vs 4.9% private; *p* < 0.001)). Furthermore, HCPs working in metropolitan regions were more likely to recommend NIPT as a first-tier test for all pregnant patients compared to HCPs in other areas (26% vs 10.5%, respectively (*p* < 0.001)).

HCPs also varied in their first trimester ultrasound referral practices for patients having NIPT as a first-tier screen: the majority (86.1%) offered an early structural anatomy scan at 11–13 weeks, but there was variation in offerings between a ‘dating’ ultrasound at 6–8 weeks and a ‘pre-NIPT’ ultrasound at 10 weeks (Table 2).

Most respondents (53%) indicated that they offer patients a choice of targeted or expanded NIPT (Table 2). Some HCPs indicated that they offer a choice based on patient history or prior increased probability of chromosomal anomalies. Several respondents indicated support for patient autonomy and informed decision-making:

*“I want them to be informed about the varying sensitivity and specificity and confirm that they truly ‘want to know’ about each condition. In my experience some patients decline testing for CNVs [copy number variants] and SCAs [sex chromosome aneuploidy] based on condition severity or poor test accuracy.”* (P352, genetic counselor)

Others indicated that the (perceived) cost difference between targeted and expanded panels was an important consideration in whether to offer this choice:

*“Additional tests cost more money, patients may not want to have all tests completed due to personal preference- especially fetal sex, or if they [the conditions] are very rare they [the patients] may not prioritize due to cost”* (P184, general practitioner)

Just over one third (37.4%) of HCPs stated they either do not offer, or infrequently offer, expanded NIPT. Reasons provided for not offering a choice related to constraints such as patient’s capacity to pay, limited time available to consult on options, only having access to one brand of NIPT, and a lack of awareness of the options available and uncertainty about their own knowledge of conditions detected in expanded NIPT. As one respondent states:

*“I just check the standard conditions, probably because I can’t counsel as thoroughly about the other conditions”* (P368, general practitioner)

The majority of providers (60.7%) refer patients to one particular brand of NIPT. The most common reasons cited include: clinical support provided by laboratories (29.7%); test performance (28.8%); convenience of blood collection (26.9%); and familiarity with the brand (24.6%). Only 4% choose a brand based on the lowest cost (Table 2).

The greatest barriers to overall access to NIPT, whether targeted or expanded, were cost (94.1%), patient awareness (31.6%), and HCPs not informing patients of the option of NIPT (26.7%).

### Knowledge of NIPT, pre-test information provision and consent

**Fig. 1:**
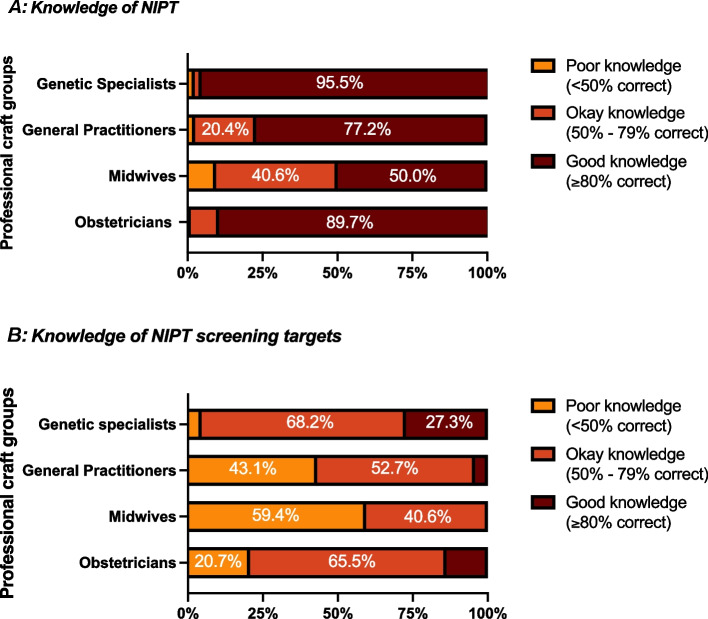
Level of knowledge by profession: **a** Knowledge about NIPT (e.g. performance, purpose, risks etc.), as assessed by the proportion of correct responses to 14 true/false statements (Supplementary Table I). **b** knowledge of what NIPT can screen for, as assessed by the proportion of correct responses to 19 true/false statements (Supplementary Table II)

The majority of participants (67.6%) reported that they felt informed or very informed about NIPT. However, the self-perception of knowledge differed significantly between professions. In an objective measure of knowledge, through a series of true/false questions, the majority of the participants (83.2%) had a good level of knowledge of NIPT (Supplementary Table 1). Knowledge levels significantly differed between professional groups (Fig. 1a; *p* < 0.001) but not by years of practice, location of practice, or sector.

However, awareness of what NIPT can be used to screen for (Supplementary Table 2; Fig. 1b) or why some things were screened varied. In regards to screening of sex chromosomes, 82.1% (*n* = 389) thought the primary purpose was for detecting sex chromosome aneuploidies, whereas 13.7% thought it was fetal sex determination and 4.2% thought there was no purpose.

**Fig. 2:**
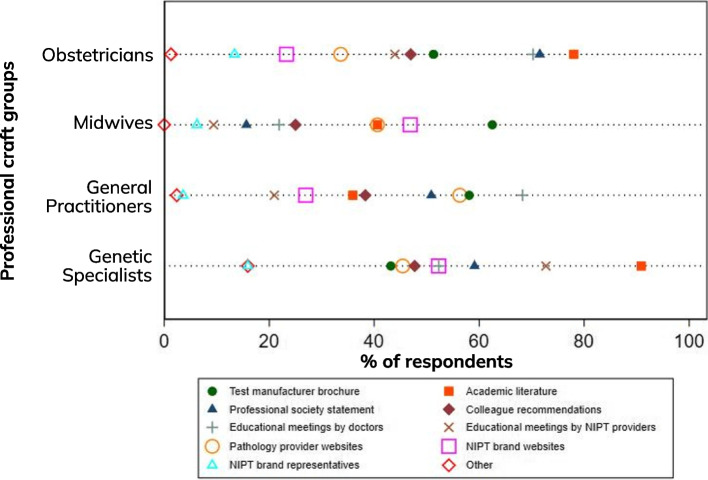
Source of information on NIPT by professional craft group

Key sources of information about NIPT also varied by professional group. Overall, professional educational meetings/workshops, academic literature and professional society statements were the most commonly reported sources of information on NIPT. The most common sources differed by profession group, with obstetricians and genetic specialists utilising the academic literature, while GPs rely on educational meetings provided by doctors. Midwives relied most heavily on test manufacturer brochures (Fig. 2).

During pretest counselling, most respondents stated they informed patients about how NIPT works (*n* = 406, 85.5%), what results are possible (*n* = 396, 83.4%), limitations of the test (*n* = 399, 84%) and how results would be returned (*n* = 357, 75.2%). Far fewer participants indicated they discussed how samples and data generated from NIPT would be stored following the test (*n* = 259, 54.5%) or incidental findings. Most respondents also provided patients with information materials including brochures/pamphlets (*n* = 286, 69.2%) and referral forms (*n* = 269, 65.1%). Just under half the sample (*n* = 192, 46.5%) also directed patients to a website.

Respondents were asked about what information patients need to know about NIPT in order to provide informed consent, reported in Table 3 below.

**Table 3:** What do patients need to know about non-invasive prenatal testing in order to give informed consent for the test

	n (%)
**NIPT (in general)**	
NIPT is a screening test and not diagnostic	431 (90.7)
What conditions can be detected with NIPT	427 (89.9)
How much NIPT costs	425 (89.5)
NIPT analyzes cell-free fetal DNA in maternal blood	355 (74.7)
NIPT could detect chromosomal changes of unknown significance	252 (53.1)
Accuracy of NIPT for each genetic condition it is screening for	238 (50.1)
The clinical presentation and prognosis of the genetic conditions NIPT can screen for	229 (48.2)
**NIPT results**	
That results may need to be confirmed with diagnostic testing	418 (88.0)
What a ‘positive’ or ‘negative’ result means	415 (87.4)
How long it takes to receive the NIPT results	376 (79.2)
How the results will be provided to the patient	369 (77.7)
What options are available following a high-probability NIPT result	352 (74.1)
What diagnostic testing involves and the associated risks	319 (67.2)
**Limitations of NIPT**	
That NIPT cannot detect all genetic conditions	419 (88.2)
Possibility of a false positive or false negative NIPT result	409 (86.1)
Possibility of receiving incomplete or no results	403 (84.8)
NIPT cannot detect all other possible causes of a condition or disability	353 (74.3)
Possibility of incidental findings	271 (57.1)
Sex of the baby cannot be withheld if an sex chromosome aneuploidy is suspected	229 (48.2)
Reasons that someone may receive incomplete or no results	207 (43.6)
NIPT results could be affected by placental mosaicism	196 (41.3)
The type of incidental findings that are possible	117 (24.6)
**Post-test management of sample and test results**	
How data from results will be managed and/or used in the future	268 (56.4)
How the blood sample will be managed and/or used in the future	159 (33.5)
How long data from results will be stored	127 (26.7)
How long the blood sample will be stored	110 (23.2)

Respondents were asked about their impressions of the overall adequacy of pre-test information provision in preparing patients for possible test results. Just over half the respondents (*n* = 232, 55.9%) thought pre-test counseling was moderately adequate in preparing patients for possible results. Almost equal proportions thought the adequacy of pre-test counseling was low (*n* = 91, 21.9%) or high (*n* = 92, 22.2%).

Respondents were asked how pre-test counseling could be improved. Many suggested a need for new or improved informational materials, such as these being accessible in varying formats (video, apps etc.) and languages. Some indicated a need for standardized, non-branded materials to aid with counseling and information provision, as well as a need for greater education of providers:

*“Online accessible information to shift the time burden from healthcare providers such as GPs.”* (P434, genetic counselor)

*“Clear non-biased [sic] handout. Provider cheat sheet with key points to cover for consent. Detailed consent form.”* (P117, general practitioner)

*“Better informed [general practitioners] and [midwives] who do early antenatal bookings - understanding the difference between cFTS and NIPT, the importance of ultrasound, the accuracy of the test, the role of diagnostic testing”* (P112, Obstetrician)

### Post-test management and outcomes following NIPT results

Of the 475 respondents, 423 (89%) indicated that they are involved in providing post-test counseling. About half (*n* = 207, 49.5%) reported that NIPT has increased their workload. Respondents described increased time and resource demands for (complex) counseling (pre- and post-test), information provision, and additional testing or specialist referrals. Conversely, 38.3% (*n* = 160) reported no change in workload, with some indicating the time required to counsel patients about NIPT has replaced that for CFTS, or a reduction in workload (*n* = 25, 6%).

Many participants emphasized the impact of time constraints on the quality of pre- and post- test counseling and described the need for more funding.

*“NIPT, and also expanded reproductive carrier screening, are complex concepts which need to be discussed well. This takes time. Current Medicare structures encourage short appointments in general practice… which are not conducive to good holistic care in this area. Effectively I take a pay cut for providing comprehensive counseling…”* (P401; general practitioner)

*“This is complex counseling and takes a long appointment in general practice. Improved… [public funding models] would encourage a better service for all pregnant patients.”* (P401, general practitioner)

However, other participants questioned whether more time would end up being useful, highlighting difficulties in meaningfully engaging patients with large amounts of complex information.

*“Having more time to do so, BUT this is almost impossible. Those with an interest in mental health, obesity, smoking, diet, food safety, exercise, etc will all advocate for more time to be spent…We may all need to spend hours of counseling with each patient, by the end of which they would be totally overwhelmed by what they’ve been told…”* (P92, obstetrician)

*“Thorough pre-test counseling is difficult to achieve, patients often don’t want a lot of information, they just want to have the test as they are expecting to get reassuring information. Even if you provide really good pre-test counseling, many won’t engage…” (P84, genetic counselor)*

*“Most patients cannot grapple with such complexities before a result. It is naive to believe you can adequately inform a non medically trained person in most cases” (P48, obstetrician)*

The majority of respondents (*n* = 387, 84.9%) reported they find it easy or very easy to interpret NIPT results received from the laboratory. 74.1% of respondents stated they receive accompanying explanatory notes and 79.3% (*n* = 361) can access laboratory staff to discuss findings if necessary. Approximately one-third of respondents (*n* = 155, 34%) indicated they would like more information or support from the laboratory than they currently receive, particularly when dealing with certain types of results (e.g. high chance, rare, incidental, incomplete, or no results) or to clarify their own understanding before returning results.

HCPs communicate NIPT results to patients in different ways, depending on the outcome of the test. Just over half the respondents (*n* = 256, 56.1%) reported that a positive result would be conveyed through a face-to-face appointment. For a negative result, or anything other than a positive result, the majority of respondents indicated patients would be informed via a phone call (54.4% and 49.3%, respectively).

Respondents were divided on the terminology used to describe the results of NIPT. For negative screen results, just over half (*n* = 234, 51.4%) describe a negative screen as a ‘low risk result’ and 36.5% (*n* = 166) use ‘low probability or chance result’. Similarly, just under half (*n* = 201, 44.7%) describe a positive screen as an ‘increased/high risk result’, and 44.4% (*n* = 200) use ‘increased/high probability or chance result’.

Antenatal care pathways after NIPT differed depending on the outcome of NIPT (Supplementary Table 3). While advice following some results (e.g. low/high probability, no call) was relatively consistent among respondents, advice varied when dealing with instances where not all requested results were returned (refer to someone else (20.2%); repeat NIPT (19.8%); diagnostic testing (15.8%)).

Despite 77.5% indicating they advise patients with a high probability result to have diagnostic testing, just over one quarter of respondents (27.7%) indicated that they believe some of their patients had terminated their pregnancy based on a high probability NIPT result, without prenatal diagnostic confirmation. Respondents elaborated via text responses under what circumstances they believed this to be occurring. Many reported this occurred when NIPT results were concordant with ultrasound or CFTS findings, or were in addition to structural anomalies identified through ultrasound. Others outlined various logistical reasons, such as advanced maternal age, or patients not wanting to wait or travel for diagnostic testing:

*“I have experienced this with 3 patients who have not felt prepared to wait for amnio and results at 18/40 before making decision on pregnancy”* (P102, genetic counsellor)

*“They do not want to travel 400km to where invasive testing can be undertaken and then wait for results (after waiting for appointment for testing).” (P125, obstetrician)*

Some participants thought the decision to terminate was influenced by how the patient understood their NIPT result. Some were concerned that patients interpreted their high probability result as diagnostic, whereas others reported that some patients understood NIPT is a screening tool but are nonetheless comfortable making a decision on the basis of the result:

*“Given the high specificity of positive NIPT, which is often understood by many patients, many assume this result to be confirmed.”* (P143, general practitioner)

*“some patients comfortable with the 98% chance (for example) that fetus [sic] will have T21, and don’t feel the need to confirm” (P71, genetic counsellor)*

Finally, just under one fifth (18.1%; *n* = 83) of participants reported that they had handled an incidental finding. In 83% of these cases, these results were accompanied by explanatory notes from the laboratory, and 87% of participants found these notes useful for interpreting the results. While the patient was informed of the finding in almost all cases (98%), 32% of this group of respondents reported that they did not feel adequately prepared to disclose the finding, and 39% would have liked more support and/or information from the laboratory than they received.

## Discussion

This study provides important insights into how NIPT has been incorporated into antenatal healthcare across Australia. In Australia, NIPT is offered by both public and private antenatal healthcare services but does not attract public funding - the test is solely available as ‘user-pays’. This particular configuration of the implementation of NIPT in Australia appears related to the several variations we report in the delivery of NIPT, which may raise concerns about equitable access and disparities in the quality of care. This is particularly the case in regard to people with fewer economic resources and those in remote and rural communities, as many HCPs are concentrated in urban areas.

The first key difference we identify is that between whether NIPT is offered as a first or second-tier test (ie. subsequent to high probability results on another screening test). Our data indicate that HCPs in private practice are more likely to adopt NIPT as a first-tier test for all pregnancies. Conversely, we found HCPs in rural areas and working solely in the public sector were less likely to offer NIPT as a first-tier test. This may be connected to the high cost of NIPT, as patients in rural and remote areas of Australia are more likely to be of lower socioeconomic status [4]. While this divergence is consistent with the position statement of RANZCOG, the Human Genetics Society of Australasia [7], and the International Society of Prenatal Diagnosis [8] which remain agnostic on whether NIPT should be a first or second-tier test, it contrasts with the recommendations by the American College of Medical Genetics and Genomics [9]. They recommend that NIPT be used as a first-tier screening tool for all pregnant persons.

Another key point of diversity in NIPT provision is whether patients are offered a range of options or tests to choose from. There are a range of commercial providers and brands of tests in Australia, with different options available. Our findings suggest that many HCPs do not consistently offer patients a choice of NIPT. When options are described to patients, these usually apply to the option of sex chromosome aneuploidies and/or fetal sex; it rarely includes microdeletions or genome-wide screening. This is important, as much literature shows that when given appropriate counseling, patients want to know more about their pregnancy rather than less. In the Netherlands Trident-2 study, for example, 78% of patients chose expanded NIPT when offered as part of a national publicly funded screening program [18].

Similarly, HCPs do not typically offer patients a choice in the brand of the test. Many of the reasons HCPs cited for preferring a particular brand of test related more to logistical and other considerations (convenience, speed, brand familiarity or service loyalty). This means that the offer of targeted or expanded tests may be somewhat secondary to whether they only refer patients to a particular brand of test. In the commercially driven system of NIPT provision in Australia, some of these considerations (eg. brand familiarity and service loyalty) may be seen as raising concerns about the impact of industry and commercial interests on clinical care and patient choice [19]. However, some of the HCPs preferences (e.g. speed of results) may also align with patient preferences [20].

A key factor underlying these disparities in the offering of tests appears to be the current cost of the test. Consistent with other studies and commentaries, cost was perceived as a major barrier to accessing and providing NIPT [1, 2, 4, 5, 10, 13, 21–23]. Many of the participants noted that the costs of NIPT were prohibitively expensive for patients, not just to access the test in general, but particularly to access expanded NIPT. Previous research has found that the additional costs of expanded testing weigh heavily on pregnant people’s decision-making around the test [13]. It is worth noting, though, that in Australia, expanded testing currently does not necessarily cost more than targeted testing. While some labs do charge more, especially for 22q microdeletion screening, others that offer genome wide screening do not charge more for this. There is no additional cost for sex chromosome screening.

HCPs’ perceptions of a patient’s socioeconomic status may impact the quality and type of care they receive [24], such as whether they are offered targeted or expanded NIPT, or NIPT at all as a first-tier test. While uptake rates are not necessarily indicative of promotion of informed choice, reducing financial barriers is essential to facilitate reproductive autonomy and equity of access [25]. Again, the Dutch Trident-2 study is salutary for illustrating the effect of cost, or the perception of cost, on patient choice and access to NIPT options [26]: of the 73,239 pregnancies included in the study, 78% elected genome-wide screening when cost was not a barrier, or perceived barrier.

Importantly, despite concerns that the use of first trimester ultrasound may decline with the increase in uptake of NIPT [27], the majority of HCPs in this survey reported that they are still offering an 11–13 week ultrasound. RANZCOG and other professional bodies recommend that all women should be offered a first trimester ultrasound for the early detection of major structural malformations, even if undertaking NIPT [7, 8, 28]. However, this adds further costs to patients as government funding in Australia does not cover the full cost of ultrasound scans for patients.

The variation we report in HCP knowledge, as well as the challenges respondents described in providing adequate pre and post-test counseling, also contribute to different offering practices amongst HCPs; an issue that is likely exacerbated by the shift to expanded NIPT. HCPs’ knowledge of NIPT directly influences how NIPT is provided to pregnant people; as noted some HCPs did not offer expanded NIPT due to their lack of knowledge of the test or screening targets. Levels of knowledge varied between the professional groups but reflected expected areas of expertise: genetic specialists and obstetricians were the most knowledgeable, followed by general practitioners and then midwives. This may also reflect the value of different sources of information and knowledge, with the reliance on test provider pamphlets and websites by some groups potentially problematic.

In general, our data support the need for improved HCP education as a critical component of high quality care in prenatal screening, and to underpin values such as patient autonomy and informed consent. Professional societies and the Nuffield Council have made recommendations as to what information should be included in pre-test counselling [7–9, 29]. While the majority of HCPs in our study agreed with most of the content recommended by these bodies, there were some notable discrepancies in the provision of information on the accuracy of NIPT, clinical presentation and prognosis of genetic conditions, and possibility of incidental findings. Respondents echoed the calls of others for more time or new or improved educational materials [9, 13, 30, 31] to support high quality counseling. Given that informational materials have previously been criticized as being biased, misleading, inaccurate and/or incomplete [29], the suggestions for standardized, non-branded materials for HCPs by our respondents seem particularly important. Such materials have been developed by government or professional institutions in other countries [32, 33]. More widespread use of balanced and standardized information materials may also support a broader use of neutral language when discussing results (e.g. “probability” or “chance” vs “risk”), which fewer than half of HCPs indicated they use currently.

Respondents also pointed to challenges in improving pre-test counseling. Patient experiences may be more dependent on the result they receive than the quality of pre-test counseling. Hence, it is vital to pay attention to the post-test counseling context and provision of adequate support for patients after return of results [34]. This is especially important for informed decision-making following a high chance result, given the concerns we report about patients interpreting their NIPT result as diagnostic and terminating pregnancies. Additional support is warranted for HCPs in returning results, particularly when dealing with uncommon results such as incidental findings.

However, the provision of more information in itself is insufficient to support HCPs and ensure that patients using NIPT receive high quality care that respects autonomy. As respondents pointed out, the additional time required for this should be recognised, potentially through increasing compensation for HCPs, for example, via the public healthcare system. Several respondents indicated that the current system disincentivizes thorough counseling, as providers are not remunerated fully for the time needed to counsel on NIPT. This suggests that any movement toward public funding of NIPT to increase availability and access will also require recognition of the impact of this on delivery of antenatal care, and include it as a critical factor in resource allocation decisions.

The strength of this study is that it is the first report of the clinical experience of NIPT in Australia from the perspectives of several different HCP groups. It builds upon previous reports that predominantly focused on the views of obstetricians and provides a more comprehensive summary of the experience of NIPT in Australia. However, as a result of the sampling method used, there are limitations to our sample. It is not necessarily representative and could be affected by self-selection bias. Nonetheless, this study provides valuable information on how NIPT has been implemented and identifies several discrepancies in NIPT provision in Australia. Further research to explore the views of HCPs who do not use NIPT as well as patient perspectives would be valuable to understanding the provision of NIPT in Australia.

## Conclusion

This study reports on the Australian experience of implementing NIPT outside of a coordinated program and provides valuable insights for other high-income nations on the successes and challenges to its provision. While NIPT is now being used widely in Australia, several persistent challenges continue to hamper clinical implementation and evaluating equity of access. Primary among these challenges is the lack of systematic population-based data collection on the uptake and type of prenatal screening. This survey suggests that significant clinical variation and inequity of access to NIPT exists. While our study does not provide definitive data to drive funding policy change, it can inform a range of possible ways for governments and/or professional bodies to improve NIPT provision, such as increase in education of HCPs, including development of standardized and non-commercial informational materials; more time and resources for counseling (e.g. increasing funding to antenatal care services); reviewing and adapting current models for informed consent; standardizing offers of types of NIPT; and decreasing cost barriers for patients. These steps would ensure that Australians continue to receive a high standard of antenatal care.

### Supplementary Information

Supplementary Material 1.

## Data Availability

The datasets used and/or analysed during the current study are available from the corresponding author on reasonable request.

## Abbreviations

NIPT: Non-invasive prenatal testing
HCP: Healthcare professional
CFTS: Combined first trimester screening
GPs: General practitioners
RANZCOG: Royal Australian and New Zealand College of Obstetricians and Gynaecologists
