# Supplementary material for: Disparities in integrating non-invasive prenatal testing into antenatal healthcare in Australia: a survey of healthcare professionals
Source: BMC Pregnancy Childbirth. 2024 May 14;24:355. doi: 10.1186/s12884-024-06565-1 (PMC11092221; doi:10.1186/s12884-024-06565-1)
Supplement: Supplementary file 1 — Supplementary Material 1. [file 12884_2024_6565_MOESM1_ESM.docx]

# Disparities in integrating non-invasive prenatal testing into antenatal healthcare in Australia: A survey of healthcare professionals

### Supplementary Table I: Healthcare professionals’ knowledge of NIPT as assessed by a series of true/false statements.

| **Knowledge statements (correct response)** | **Correct response** |
| --- | --- |
|  | **n(%)** |
|  |  |
| NIPT is diagnostic (F) | 433 (91.2) |
| NIPT is 100% accurate (F) | 463 (97.5) |
| NIPT has a risk of miscarriage (T) | 465 (97.9) |
| NIPT tests for all genetic conditions (F) | 467 (98.3) |
| NIPT has many brand names but they all screen for the same conditions (F) | 321 (67.6) |
| NIPT is more accurate for detecting Down Syndrome than combined first-trimester screening (T) | 451 (95.0) |
| NIPT has the same level of accuracy for all the conditions it screens for (F) | 418 (88.0) |
| A ‘low probability’ or ‘low risk’ result means everything is okay with the fetus (F) | 462 (97.3) |
| NIPT can determine the fetal sex (T) | 465 (97.9) |
| NIPT will always return a result (F) | 449 (94.5) |
| NIPT is usually available from 10 weeks gestation onwards (T) | 466 (98.1) |
| Maternal factors can affect the chance of getting a result on NIPT (T) | 435 (91.6) |
| NIPT can find out things about the pregnant person's health (T) | 205 (43.2) |
|  |  |
| **Total knowledge score – proportion of correct response** |  |
| ≥ 80% correct = good knowledge (>10 correct statements) | 395 (83.2) |
| 50-79% correct = okay knowledge (7-10 correct statements) | 70 (14.7) |
| <50% correct = poor knowledge (≤ 6 correct statements) | 10 (2.1) |

###

### Supplementary Table II: Healthcare professionals’ knowledge of screening options currently available through NIPT, as assessed by a series of true/false statements.

| **NIPT screening target (correct response)** | **Correctly identified n(%)** |
| --- | --- |
| Trisomy 21 (Down Syndrome) (T) | 468 (98.5) |
| Trisomy 13 (Patau Syndrome) (T) | 460 (96.8) |
| Trisomy 18 (Edwards Syndrome) (T) | 461 (97.1) |
| Rare autosomal trisomies (T) | 186 (39.2) |
| 47,XXY (Klinefelter Syndrome) (T) | 402 (84.6) |
| 45,X (Turner Syndrome) (T) | 414 (87.2) |
| 47,XXX (Triple X Syndrome) (T) | 387 (81.5) |
| 47,XYY (Jacobs Syndrome) (T) | 380 (80.0) |
| Prader-Willi Syndrome (T) | 119 (25.1) |
| Angelman Syndrome (T) | 111 (23.4) |
| Cri-du-Chat Syndrome (T) | 115 (24.2) |
| 22q11.2 Deletion Syndrome (DiGeorge Syndrome) (T) | 310 (65.3) |
| Certain single-gene conditions (T) | 92 (19.4) |
| Fetal sex (T) | 435 (91.6) |
| Triploidy (T) | 199 (41.9) |
| Identical vs non identical twins (T) | 52 (10.9) |
| Unbalanced translocations (T) | 99 (20.8) |
| Large copy number variants (T) | 97 (20.4) |
| Genome-wide screening (T) | 86 (18.1) |
|  |  |
| **Total knowledge score – proportion of correct response** |  |
| ≥ 80% correct = good knowledge (>15 correct) | 51 (10.7) |
| 50-79% correct = okay knowledge (9-15 correct) | 283 (59.6) |
| <50% correct = poor knowledge (≤ 8 correct) | 141 (29.7) |

### Supplementary Table III: Antenatal care pathways advised following non-invasive prenatal testing outcomes

|  | **n(%)** |
| --- | --- |
| **Advice to a patient receiving low probability result** |  |
| No additional investigations and continue antenatal care | 348 (73.3) |
| Repeat NIPT | 7 (1.5) |
| Other aneuploidy screening test e.g. CFTS | 32 (6.7) |
| Diagnostic testing e.g. CVS or amniocentesis | 2 (0.4) |
| Refer to someone else | 4 (0.8) |
| Other | 85 (17.9) |
| I have never had to manage this situation | 8 (1.7) |
| **Advice to a patient receiving high probability result** |  |
| No additional investigations and continue antenatal care | 7 (1.5) |
| Repeat NIPT | 2 (0.4) |
| Other aneuploidy screening test e.g. CFTS | 18 (3.8) |
| Diagnostic testing e.g. CVS or amniocentesis | 368 (77.5) |
| Refer to someone else | 196 (41.3) |
| Other | 49 (10.3) |
| I have never had to manage this situation | 10 (2.1) |
| **Advice to a patient who did not receive a result following NIPT** |  |
| No additional investigations and continue antenatal care | 8 (1.7) |
| Repeat NIPT | 339 (71.4) |
| Other aneuploidy screening test e.g. CFTS | 118 (24.8) |
| Diagnostic testing e.g. CVS or amniocentesis | 67 (14.1) |
| Refer to someone else | 38 (8.0) |
| Other | 67 (14.1) |
| I have never had to manage this situation | 45 (9.5) |
| **Advice to a patient if NIPT returns a maternal incidental finding** |  |
| No additional investigations and continue antenatal care | 8 (1.7) |
| Repeat NIPT | 22 (4.6) |
| Other aneuploidy screening test e.g. CVS or CFTS | 26 (5.5) |
| Diagnostic testing e.g. CVS or amniocentesis | 28 (5.9) |
| Refer to someone else | 180 (37.9) |
| Other | 62 (13.1) |
| I have never had to manage this situation | 194 (40.8) |
| **Advice to a patient not all requested results are reported** |  |
| No additional investigations and continue antenatal care | 33 (6.9) |
| Repeat NIPT | 94 (19.8) |
| Other aneuploidy screening test e.g. CVS or CFTS | 38 (8.0) |
| Diagnostic testing e.g. CVS or amniocentesis | 75 (15.8) |
| Refer to someone else | 96 (20.2) |
| Other | 90 (18.9) |
| I have never had to manage this situation | 140 (29.5) |
